# *Clostridioides difficile* evolution in a tertiary German hospital through a retrospective genomic characterization

**DOI:** 10.1007/s15010-025-02576-y

**Published:** 2025-06-08

**Authors:** Fabian Lorenzo-Diaz, Tilman E. Klassert, Cristina Zubiria-Barrera, Amelya Keles-Slevogt, Mario Gonzalez-Carracedo, Mariano Hernandez, Hortense Slevogt, Thomas Grünewald

**Affiliations:** 1 Genomics and Health Group, Departamento de Bioquímica, Microbiología, Biología Celular y Genética, Facultad de Ciencias, Canary Islands, Universidad de La Laguna, La Laguna, Spain; 2 Instituto de Enfermedades Tropicales y Salud Pública de Canarias, Canary Islands, Universidad de La Laguna, La Laguna, Spain; 3 Department of Respiratory Medicine and Infectious Diseases, Hannover Medical School, German Centre for Lung Research (DZL), BREATH, Hannover, Germany; 4 Dynamics of Respiratory Infections Group, Helmholtz Centre for Infection Research - HZI Braunschweig, Braunschweig, Germany; 5 Department of Neurology and Experimental Neurology, Charité - Universitätsmedizin Berlin, Berlin, Germany; 6 German Center for Neurodegenerative Diseases (DZNE) Berlin, Berlin, Germany; 7 Clinic for Infectious Diseases and Tropical Medicine, Klinikum Chemnitz, Chemnitz, Germany; 8 Present Address: Department of Infectious Diseases and Nephrology, Klinikum St. Georg, Leipzig, Germany

**Keywords:** Clostridioides difficile, CDI, PaLoc, WGS, Antibiotic resistance genes

## Abstract

**Purpose:**

*Clostridioides difficile* is a major cause of healthcare-associated infections, contributing to significant morbidity and mortality. This study aimed to investigate the genomic characteristics, antimicrobial resistance (AMR) profiles, and temporal dynamics of *C. difficile* strains isolated from hospitalized patients in a German tertiary hospital over nearly two decades (1997–2015).

**Methods:**

Whole-genome sequencing was performed on 46 toxigenic *C. difficile* isolates to determine sequence types (STs) and phylogenetic relationships and these were compared to national surveillance data on *C. dificile*. AMR profiling was conducted to identify key resistance determinants at genetic level while epsilometer minimum inhibitory concentration (MIC) analyses were used to correlate genetic resistance markers with phenotypic resistance. Longitudinal antibiotic usage data were analysed to assess potential associations with resistance profiles and strains evolution.

**Results:**

Five predominant STs were identified: ST1 (30%), ST54 (24%), ST3 (22%), ST11 (11%), and ST37 (4%). Phylogenetic analysis showed that ST1 (ribotype 027) emerged as the dominant and persistent lineage, replacing ST11 and ST54 over time. AMR profiling detected several resistance genetic markers such as *CDD-1/CDD-2* (carbapenem resistance), *ErmB* (macrolide-lincosamide-streptogramin B resistance/MLS resistance), and mutations in *gyrA* (fluoroquinolone resistance) and *rpoB* (rifampicin resistance). MIC analyses confirmed high resistance rates to moxifloxacin (87%) and rifampicin (59%), while susceptibility to fidaxomicin, metronidazole, and vancomycin remained. The *tetM* gene, associated with doxycycline resistance, declined as ST11 and ST54 frequencies decreased. Longitudinal analysis revealed a reduction in moxifloxacin resistance following its decreased use, whereas increased doxycycline use paradoxically correlated with reduced resistance.

**Conclusion:**

This study highlights the dynamic strain evolution of *C. difficile*, reflecting national trends in strain evolution. The findings emphasize the strong correlation between epsilometer MIC values and molecular resistance markers. This observation reinforces the integration of genetic surveillance with antibiotic stewardship in the clinical routine to effectively mitigate CDI recurrence. Further research is needed to better understand the complex interactions between antibiotic exposure and strain evolution in hospital environments.

**Supplementary Information:**

The online version contains supplementary material available at 10.1007/s15010-025-02576-y.

## Introduction

*Clostridioides difficile* is the leading cause of hospital-acquired diarrhoea and has emerged as a significant global public health concern over the past decades [1]. As a Gram-positive, anaerobic bacillus capable of sporulation, it can persist on surfaces for extended periods, facilitating its dissemination and transmission within and beyond hospital environments [2]. According to the European Centre for Disease Prevention and Control (ECDC), *C. difficile* was the sixth most frequently detected microorganism in European acute care hospitals between 2016 and 2017, causing 189,526 healthcare-associated *C. difficile* infections (CDI) annually, with an associated mortality of 7,864 patients (ECDC, 2023). A multicenter study involving 37 German hospitals estimated the annual economic burden of CDI at €464 million, with the first CDI episode costing between €5,798 and €11,202 per patient [3]. Additionally, recurrent CDI cases required an average hospital stay of 55 days, incurring costs of €52,024 per patient, as reported by the COMBACTE-CDI consortium [4].

Antibiotic-induced disruption of the gut microbiota, particularly in immunosuppressed patients, is the primary risk factor for CDI [5]. Intestinal symptoms are linked to the Pathogenicity Locus (PaLoc), which harbors toxin-coding genes *tcdA* and *tcdB* that compromise the integrity of intestinal epithelial cells [6]. Furthermore, the Binary Toxin Locus (CDT), comprising *cdtA* and *cdtB*, has been implicated in bacterial adhesion, colonization, and colonic inflammation [7]. Although multiple treatment strategies have been proposed, standard CDI therapy relies on antimicrobial administration [1]. Historically, metronidazole was the first-line treatment for non-severe CDI. However, since 2021, the European Society of Clinical Microbiology and Infectious Diseases (ESCMID) no longer recommends its use when fidaxomicin is available [8]. CDI management has become increasingly challenging due to antimicrobial resistance (AMR), often mediated by genetic polymorphisms or mobile genetic elements. Frequent genetic exchange in *C. difficile* contributes to genomic plasticity and enhances adaptation to hospital environments [1, 6].

The determination of molecular markers such as ribotypes (RT) or sequence types (ST) have facilitated CDI epidemiological studies, tracking the spread of hypervirulent strains across countries. A CDI surveillance study in Germany (2019–2021) identified RT014 (ST2/14), RT001 (ST3), and RT078 (ST11) as the most prevalent lineages [9]. These findings aligned with ECDC epidemiological reports from 2016 to 2017, which also identified RT014 as the most common ribotype in Europe, with a prevalence of 17% (ECDC, 2022). In previous years however, the EUCLID study reported RT027 as the most prevalent strain in Europe between 2012 and 2013 (19%), followed by RT001 and RT014 [10]. A similar ribotype distribution was also reported by an epidemiologic study in Germany between 2014 and 2019 [11]. All these findings suggest temporal shifts in *C. difficile* lineage prevalence, with RT027 exhibiting a steady decline since 2016 and being detected only sporadically from 2019 to 2021 [9, 11].

RT027 (ST1) was first identified in North America in 2002 and was associated with large-scale outbreaks of hypervirulent strains [12]. In Germany, the first RT027 cases were reported in 2007 in hospitals across the southwestern regions [13]. Whole-genome sequencing has been instrumental in reconstructing the spatial and temporal dynamics of RT027, including a study analyzing 57 isolates collected from 36 German locations between 1990 and 2012 [14]. The present study aimed to leverage genomic sequencing technology to analyze 46 toxigenic *C. difficile* strains collected from hospitalized patients at St. Georg Hospital (Leipzig, Germany) over an 18-year period (1997–2015). The study assessed the presence of hypervirulent RT027 and other virulent ribotypes, antibiotic resistance profiles, and the impact of hospital antibiotic usage on *C. difficile* evolution. By sequencing strains over an extended period, this research aimed to elucidate strain evolution, antibiotic resistance profiles, and the potential emergence of hypervirulent lineages.

## Materials and methods

### Biological samples and study design

Liquid stool samples were tested for the presence of *C. difficile* by direct toxin detection and/or bacteriological culture with in-vitro toxin production by antigen test using Ridascreen™ assay (R-Biopharm, Germany). Specimen were taken into culture on Columbia Blood Agar (bioMérieux, France) and on a selective *C. difficile* Agar (Heipha, Germany). A more sensitive chromogenic agar (bioMérieux, France) was used for samples after 2013. After incubation under anaerobic conditions for 48 h, bacteria were resuspended in 0.9% saline, aliquoted for antimicrobial susceptibility testing and stored on microbank™ (Pro-Lab Diagnostics, UK) at -70 °C for further investigations. A total of 46 strains covering the years 1997 and 1998, as well as the years 2008–2015, could be recovered for whole-genome sequencing. No patient data were used for this analysis; thus, no additional approvals were necessary. All methods were performed in accordance with relevant guidelines and regulations.

### Antibiotic consumption and resistance testing

Hospital-wide antimicrobial consumption data (available for 1999–2015) in the respective time series were collected in order to test their potential effect on resistance patterns observed across the study. To account for temporal effects, the mean recommended daily doses (RDD) from the previous three years were recorded for each specific year. For resistance testing, isolates were resuspended in 0.9% saline solution creating a suspension with defined turbidity (McFarland standards 1.0) and screened for resistance to metronidazol, vancomycin, moxifloxacin, rifampicin, doxycyclin and daptomycin using epsilometer strips (bioMérieux, France), following the protocol described by the manufacturer. Inhibition ellipses were examined at 48 h on blood agar plates. Minimum inhibitory concentration (MIC) breakpoints were adapted from the EUCAST V15.0 guidelines for vancomycin (2 µg ml − 1) and metronidazole (2 µg ml − 1); ECOFF values from EUCAST V11.0 for rifampicin (0.004 µg ml − 1), daptomycin (4 µg ml − 1) and fluoroquinolones (4 µg ml − 1), and from the Clinical & Laboratory Standards Institute (CLSI) guidelines for doxycycline (2 µg ml − 1).

### DNA extraction and whole-genome sequencing

Genomic DNA was extracted with the InnuPrep Bacteria DNA kit (AnalitikJena, Germany). A total of 500 ng of DNA was used as input for barcoded-library preparations with the Ion Xpress Plus Fragment Library kit (Thermo Fisher Scientific, Inc., USA). Clonal amplification and P1 Ion semiconductor chip loading were performed in the IonChef platform (Thermo Fisher Scientific, Inc., USA). Pooled samples were sequenced in an Ion Proton sequencer (Thermo Fisher Scientific, Inc., USA), using the HiQ 200-bp sequencing chemistry. Quality control and raw data processing was carried out with the PrinSeq software (Schmieder and Edwards, 2011). Sequencing reads were filtered by a minimum length (100 bp) and quality (> Q20, 5 bp sliding window). Sequencing data were submitted to the Sequence Read Archive (SRA) and assigned study accession number PRJNA1234841.

### Genome assembly and annotation

The online IonGAP platform was used to perform genome assembly and subsequent analyses [15]. Input fastq files were subsampled to 3 million reads by using the *seqtk_sample* tool, and assembled under the default options. The *C. difficile* 630 genome (NC_009089.1) was provided as reference to perform the comparisons and annotate genetic variants. Taxonomic classification was executed in IonGAP through BLASTN alignments against the *16 S rRNA* gene sequence. Multilocus Sequence Typing (MLST) profile was defined on the PubMLST webpage (http://pubmlst.org/cdifficile/). Toxins genes were obtained from the IonGAP classification and annotation module. In silico antimicrobial resistance profiling was performed with the Resistance Gene Identifier tool available from the CARD server [16].

### Phylogenetic and statistical analysis

GFF files were obtained with Prokka for the 46 *C. difficile* strains, and pan-genome reconstruction was carried out using Roary, at the Galaxy Server (The Galaxy Community, 2022). A core genome containing 647 genes was obtained, with a minimum identity of 95% for BLASTP, and present in at least 99% of isolates. For phylogenetic analysis, a Maximum Likelihood tree was constructed at the Galaxy Server with RaxML v7.7.6, including 1000 bootstrap replicates and using the GTR + G nucleotide substitution model. Phylogenetic tree was visualized with FigTreev1.4.4 (https://github.com/rambaut/figtree). Statistical analyses were performed in R, using a *p*-value < 0.05 to declare statistical significance.

## Results and discussion

### Genomic characterization of *the C. difficle* strains analyzed

A total of 46 *C. difficile* clinical isolates were identified from stool samples of patients with diarrhoea (**Supplementary Table ****S1**). Genome sequencing of the *C. difficile* isolates generated an average of 2,556,190 (± 673,543) raw reads per sample (mean read length of 158 ± 8 bp). A total of 120 ± 32 contigs (> 500 bp) were assembled, with an N50 length of 113.6 ± 39.1 Kb. The average assembly size and total gene counts were 4.13 Kb (70X depth coverage) and 4,154 (± 399), respectively. These results fitted to the *C. difficile* 630 reference genome (NCBI taxonomy ID: 272563), comprised by a circular chromosome of 4,290,252 bp and 3,776 predicted coding sequences [17].

Multilocus-sequence typing revealed different groups, 91% belonging to five STs. ST1 (clade 2, RT027) was the most common type, accounting for 30% of all the isolates. In our sample set, this ribotype was isolated in 2010 for the first time, which is in agreement with other studies reporting appearance of ST1 in this same year [18], even though the first identification of RT027 in Germany was already reported in 2007 [13]. The second most frequent ribotype was ST54 (24%; clade 1, RT012), followed by ST3 (22%; clade 1, RT001), ST11 (11%; clade 5, RT078), and ST37 (4%; clade 4, RT017). The remaining STs were represented by only one isolate (**Supplementary Table ****S1**).

The presence of toxin genes was evaluated for each isolate based on their genome sequence assemblies. All isolates were identified as toxigenic, as they contained at least one toxin gene. Specifically, in the PaLoc region, both enterotoxin *tcdA* and cytotoxin *tcdB* were consistently found in all samples, except for isolate Cd22 (ST11), which solely possessed the *tcdA* gene (**Supplementary Table ****S1**). Additionally, the CDT region, containing the binary toxin genes *cdtA* and *cdtB*, was identified in 89% of the isolates. The five isolates lacking the *cdtA*-*cdtB* binary toxin genes were attributed to ST3, ST37, ST54, or other less represented STs (**Supplementary Table ****S1**). This observation highlights that ST1 (RT027) was the only sequence type consistently exhibiting a complete PaLoc and CDT toxin profile across all sequenced genomes reinforcing its hypervirulent nature and dominance in *C. difficile* infections.

### Phylogenetic relationships between clinical isolates

**Fig. 1:**
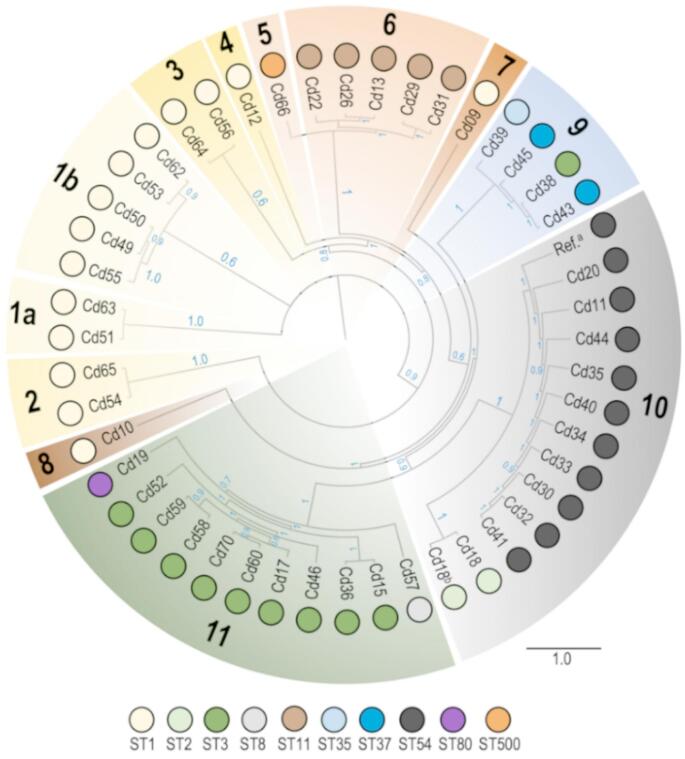
ML phylogenetic tree reconstructed from a core-genome (647 genes) for the *C. difficile* strains included in the study. Bootstrap values (1000 replicates) are shown in blue. Different statistically supported clades (1–11) were also identified with different colors. Branch length are proportional to evolutionary differences, according to GTR + G model. Sequence Type (ST) assigned to each strain by MLST analysis was also included, as colored circles. ^a^Reference sequence corresponds to *C. difficile* 630 (ST54). ^b^Isolate Cd18 was sequenced and analyzed in duplicate

Phylogenetic relationships were reconstructed with genomic sequencing data, showing a high correlation with the information provided by the sequence types. The analysis revealed a well-resolved tree topology, with all branches supported with bootstrap values higher than 60% (38 out of 45 supported by more than 80% of the replicates) (Fig. 1). The most basal groups (Clades 1a and 1b) contain seven *C. difficile* isolates, and all of them belong to ST1. Clade 2 was then diversified from the others, and Clade 3 was subsequently segregated. Next, Cd12 isolate was separated from the rest, as the unique representative of Clade 4. Interestingly all these clades are represented by *C. difficile* strains from ST1 (RT027), thus indicating this Sequence Type could be the ancestor of all *C. difficile* isolates found at the hospital. However, a clear strain replacement was observed, as Clades 5 (ST500) and 6 (ST11), were then segregated from the rest. The next two differentiation events, which corresponds to Clades 7 and 8, respectively, also include ST1 strains, therefore confirming the prevalence of this strain, as a minor representative. Clade 9 was then isolated, and it contains specific sequence types (ST35 and ST37), but also one representative of ST3, one of the most predominant strains found at the more recent clades. Clade 10 include all ST54 isolates, as well as the unique ST2 strain found in this work (Cd18). On the other hand, Clade 11 included almost all ST3 strains, as well as the unique representatives of ST80 and ST8. Overall, these results support that the most ancient *C. difficile* strains belong to ST1, from which first ST80 emerged, followed by the most recent strains, ST54 and ST3. During this period, different strains were also detected, but with a minor representation. ST11 was only detected in isolates from the first period of the study (1997-98) and overlapped with ST54 (which was present until 2010). Isolates belonging to ST1 and ST3 appeared in 2008 and coexisted in the hospital, at least until 2015.

Comparison of the strain evolution and succession in this study with national surveillance data confirms that the St. Georg Hospital follows the broader trend observed in Germany: RT027 expanded after 2007, peaked at 36% (2014–2019), and declined to 4% (2019–2021), paralleling fluoroquinolone restrictions. Similarly, RT001 (ST3) decreased from 35% (2011–2013) to 7% (2019–2021), while RT014 emerged as the dominant strain in 2022 (17%), reflecting ongoing epidemiological shifts [19].

### AMR genetic profiles

The extent of molecular markers for AMR was evaluated from whole genome sequences throughout the Resistance Gene Identifier (CARD) tool in all *C. difficile* isolates (**Supplementary Table ****S2**). All isolates encoded *CDD-1* or *CDD-2* (carbapenem resistance), *qacG* (disinfecting agents and antiseptics resistance) and a mutation in the *23 S rRNA* (C656T) that confers the resistance to macrolides and lincosamides (e.g., erythromycin and clindamycin). Most of them (> 80%) also carried *ErmB* (resistance to MLS), the *vanG* cluster genes (glycopeptide resistance), and a non-synonymous mutation (p.T82I) at the *gyrA* gene (fluoroquinolones). Different non-synonymous mutations, associated with high-level rifampicin resistance, were identified for the *rpoB* gene (amino acid changes S485P, H502Y, H502N, R505K, and I548M) in 54% of the isolates. Such high rates of rifampicin ARGs could indicate increased selective pressure from antimicrobial use in the hospital. Importantly, it might also represent an increased risk of CDI among patients groups treated with rifampicin, such as tuberculosis patients [20], or patients with osteoarticular infections [21]. Aminoglycoside (*AAC(6’)-Ie-APH(2’’)-Ia*, *aad(6)*, and *APH(3’)-IIIa*) and tetracycline (*tetM*) resistance genes were identified in 19 and 10% of the isolates, respectively. Previous analyses have reported that erythromycin and tetracycline resistance genes were the most representative AMR markers identified through genome sequencing [22, 23]. Others such as *catP*, *sat-4*, *clcD*, *mefH*, and *nimJ* were detected in a reduced number of isolates. However, none of the isolates analysed in this study harbours the *marR* or *rpoB* genetic variants (amino acid changes N1073R or V1143D/G/F), which have been associated with fidaxomicin resistance [24, 25]. In this regard, the *Clos*ER study reported that all isolates were susceptible to fidaxomicin, with scarce resistance to metronidazole and vancomycin [26].

### Correlation between epsilometer MIC and molecular markers

The phenotypic resistance showed a high correlation with the AMR genotyping (Table 1). Only four isolates were susceptible to all six antimicrobial agents tested, whereas multiple phenotypic resistance patterns were found in 67.4% of the C. *difficile* isolates analyzed, being four of them resistant to moxifloxacin and doxycycline, 17 resistant to moxifloxacin and rifampicin, and 11 resistant to moxifloxacin, rifampicin and doxycycline.

All isolates analysed were susceptible to daptomycin, exhibiting susceptibility to low antibiotic concentrations (all MICs ≤ 1 µg · ml^− 1^), and none of them was found to harbor daptomycin resistance genes. The resistance pattern to metronidazole was also identical for all *C. difficile* isolates (MICs < 2 µg · ml^− 1^), although one of them contained the *nimJ* gene (isolate Cd40/ST54). Different *nim* genes have been reported in nitroimidazole-resistant isolates of *Helicobacter pylori* and *Bacteroides fragilis* [27], but these genes have not been identified in *C. difficile* before [28]. Regarding vancomycin genotypic resistance, all *C. difficile* isolates encoded a *vanG*-type gene cluster (*vanG*_Cd_) which has demonstrated to be cryptic in vancomycin-sensitive strains [29].

**Table 1:** Genotype to phenotype antibiotic resistance correlation among *C. difficile* isolates (*n* = 46)

			Susceptible isolates				Resistant isolates		
Antibiotic	Genotype^a^		Number of isolates	Gene counts	Genotype-phenotype correlation (%)		Number of isolates	Gene counts	Genotype-phenotype correlation (%)
Moxifloxacin	*gyrA(T82I)*,* cdeA*		6	1	83,3		40	38	95,0
Rifampicin	*rpoB variants*		18	0	100		28	25	89,3
Doxycyclin	*tet(M)*		29	3	89,7		17	16	94,1
Metronidazol	*nimJ*		46	1	97,8		0	NA	NA
Vancomycin	*vanG cluster*		46	41	10,9		0	NA	NA
Daptomycin	ND		46	0	100		0	NA	NA

^a^See Supplementary Table S2 for full description of resistance gene (or gene variants) profiles. ND: Non-detected (all isolates analyzed were susceptible to daptomycin and none of them was found to harbors daptomycin resistance genes). NA: not applicable.

Resistance to moxifloxacin was detected in 87% of the isolates, with a MIC ≥ 4 µg ml^− 1^ (MIC levels in susceptible strains from 0.047 to 0.75 µg · ml^− 1^). Of those, 95% of the isolates harboured one or two genetic markers related to moxifloxacin resistance, the non-synonymous variant p.T82I at the gyrase subunit A (*gyrA*) gene and the *cdeA* efflux transporter coding-gene (**Supplementary Table S2**). Only one case (isolate Cd20/ST54) was genetically classified as susceptible to moxifloxacin (MIC = 0.38 µg · ml^− 1^) but having a copy of the *cdeA* gene (**Supplementary Table ****S1**).

Fifty-nine percent of the isolates showed resistance to rifampicin (MIC range 0.064–32 µg · ml^− 1^). Different tandems of point mutations related to dual non-synonymous variants in the beta subunit of the RNA polymerase *rpoB* gene (S485P/H502Y, H502N/R505K or R505K/I548M) were detected in almost all isolates (**Supplementary Table ****S2**). In the other hand, in none of the rifampicin susceptible isolates (all MIC ≤ 0.001 µg · ml^− 1^) *rpoB* gene mutations were detectable, showing a perfect genotype to phenotype correlation (Table 1). In the case of doxycycline, 37% isolates were classified as resistant (MIC 4–12 µg · ml^− 1^). The remaining 63% of the isolates exhibited very low MICs (0.001–0.38 µg · ml^− 1^).

The *tetM* gene was detected in the genome of isolates exhibiting high-resistance levels. Some of these isolates also harboured additional copies of other tetracycline resistance genes (*tetW*, *tet40*, *tet44*). Only one case (isolate Cd22/ST11) lacking *tetM* gene displayed phenotypic resistance to doxycycline (MIC = 12 µg · ml^− 1^) (Table 1). Contrary, three strains (isolates Cd70/ST3, Cd13/ST11 and Cd66/ST500) were phenotypically susceptible to doxycycline (MICs of 0.016, 0.025 and 0.38 µg · ml^− 1^, respectively) but having a copy of the *tetM* gene (**Supplementary Table ****S1**).

In conclusion, our data confirm a strong correlation between phenotypic antimicrobial resistance and genotypic AMR markers in *C. difficile*, particularly for fluoroquinolones, rifampicin and doxycycline. While all isolates remained susceptible to fidaxomicin, metronidazole, and vancomycin, the detection of *nimJ* in one isolate highlight the potential for emerging resistance mechanisms. The cryptic nature of *vanG* in vancomycin-sensitive strains further emphasizes the complexity of AMR evolution in *C. difficile*.

### Impact of antimicrobial hospital usage on *C. difficile* prevalence

**Fig. 2:**
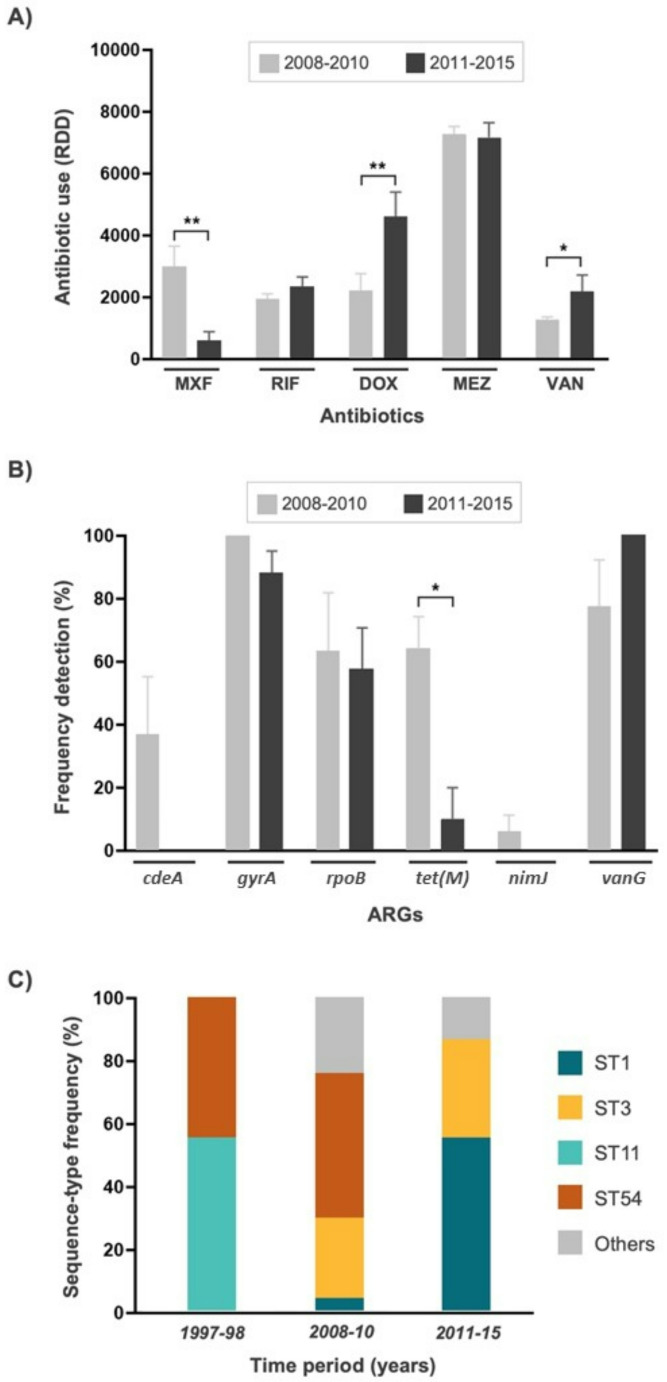
Temporal evolution of CDI cases at the St. George Hospital. (**A**) Antibiotic recommended daily dosages (RDD) applied at the St. George Hospital between 2008-10 and 2011-15 time periods. (**B**) Frequency of antibiotic resistance genes (ARG) detected in *C. difficile* isolates between 2008-10 and 2011-15. (**C**) Sequence-type (ST) frequency by time-periods. Asterisks indicate level of statistical significance (*p < 0.05, ** *p* < 0.01)

Recommended daily dosages (RDD) for moxifloxacin, rifampicin, doxycyclin, metronidazol, and vancomycin were registered between 2008 and 2015 (**Supplementary Fig. ****S1**). The use of rifampicin and metronidazole was constant over the time. Moxifloxacin was mainly used during 2008–2010, with a significant reduction in the 2011–2015 period (Fig. 2A). In contrast, both vancomycin and doxycycline use significantly increased in 2011–2015 with respect to the 2008–2010 period (Fig. 2A). Distribution of genetic AMR molecular markers and STs along the same two-time periods denoted a significant decay in the number of isolates harboring the *tetM* gene (Fig. 2B), accompanied by an increase in the presence of isolates belonging to ST1 and ST3 (Fig. 2C). These results indicated that the evolution of *C. difficile* strains at the hospital was mainly characterized by a substitution of ST11/ST54 for ST1/ST3 linages in the time period studies. Given that *tetM* presence was found to be intrinsically correlated to ST11 and ST54, but was not detected in the ST1 and ST3 isolates (**Supplementary Table ****S2**), doxycycline resistance levels were drastically reduced in the last period of the study. However, this replacement of *C. difficile* STs does not seem to be associated with an increase in the doxycycline RDD over time inside the hospital. Given that *tetM* is linked to the *Tn*5397 [6], this result might be explained by strain replacement rather than horizontal gene transfer between isolates. Thus, the observed strain turnover might suggest a combination of endemic persistence and external introductions, indicating that St. Georg follows national CDI dynamics.

In summary, by analyzing *C. difficile* at a single hospital over nearly two decades, we were able to track a granular perspective of strain turnover, resistance development, and the direct effects of antibiotic policies in a controlled setting. Unlike national surveillance studies, which aggregate data from multiple hospitals, our longitudinal, high-resolution dataset provides crucial insights into how *C. difficile* adapts to local antibiotic pressures and infection control measures over time in a real-world clinical setting. Thereby our study showed that even single centre studies may reflect broader national trends in strain evolution and antibiotic resistance profiles. Genomic analyses unveiled a diverse population primarily dominated by ST1 (RT027), although a transition from ST11/ST54 to ST1/ST3 lineages was observed over time. Prevalent resistance to moxifloxacin and rifampicin was observed, with specific genetic markers, such as mutations in the *gyrA* gene and the presence of the *cdeA* efflux transporter gene, strongly correlating with resistance levels. Notably, alterations in recommended daily dosages (RDD) of moxifloxacin, rifampicin, and doxycycline correlated with shifts in CDI prevalence and resistance profiles. Particularly noteworthy was the significant reduction in moxifloxacin usage from 2008 to 2010, contrasted with the substantial increase in vancomycin and doxycycline usage from 2011 to 2015. While the antibiotic RDDs and the ARG frequencies correlated for certain antibiotics such as moxifloxacin, the opposite was observed for doxycycline. Despite heightened doxycycline usage, a decrease in doxycycline resistance was observed, suggesting that additional factors beyond antibiotic exposure contribute to the evolution and dissemination of *C. difficile* strains in hospital settings.

While this study provides valuable insights into the evolution and antimicrobial resistance profiling of *C. difficile* in a clinical setting, certain limitations have to be acknowledged. These include the temporal gaps in sampling (i.e. missing years) and the relatively limited number of collected samples. Although the sample size may appear modest, the number of sequenced isolates (*n* = 46) is consistent with the sample sizes reported for particular regions in comparable molecular epidemiological studies [11]. A distinguishing strength of our study, however, lies in its concentrated focus on a single tertiary care hospital, enabling a high-resolution analysis of *C. difficile* strain dynamics within a stable clinical environment. Despite the aforementioned limitations, the selected isolates offer a well-distributed and representative snapshot of temporal trends during the study period, particularly surrounding a major shift in local antimicrobial guidelines implemented in 2011.

## Conclusions

This study investigates the dynamic strain evolution of *C. difficile* from a granular perspective in a single health care centre. Interestingly, the findings demonstrate that localized strain surveillance can closely reflect national trends in strain turnover. Furthermore, the resistance profiling revealed a strong correlation between phenotypic and genotypic testing, underscoring the value of integrating genetic surveillance into routine clinical practice to support antibiotic stewardship and reduce the risk of CDI recurrence. Interestingly, however, resistance profiles from our dataset did not always correlate with the antibiotic RDDs changes over time. Further research is needed to better understand the complex interactions between antibiotic exposure and strain evolution in hospital environments.

## Electronic supplementary material

Below is the link to the electronic supplementary material.

Supplementary Material 1

## Data Availability

Sequencing data were submitted to the Sequence Read Archive (SRA) and assigned study accession number PRJNA1234841. All analysis performed was solely conducted on bacterial strains and hospital-wide consumption data. No data from humans were accessed. In accordance with the Declaration of Helsinki, no IRB clearance was necessary for this research. Ethics, Consent to Participate, and Consent to Publish declarations: not applicable.
