# Supplementary material for: Clostridioides difficile evolution in a tertiary German hospital through a retrospective genomic characterization
Source: Infection. 2025 Jun 8;53(5):2209–18. doi: 10.1007/s15010-025-02576-y (PMC12460453; doi:10.1007/s15010-025-02576-y)
Supplement: Supplementary file 1 — Supplementary Material 1 [file 15010_2025_2576_MOESM1_ESM.pdf]

Supplementary information

Clostridioides difficile Evolution in a Tertiary German Hospital through a Retrospective Genomic Characterization

Fabian Lorenzo-Diaz, Tilman E. Klassert, Cristina Zubiria-Barrera, Amelya Keles-Slevogt, Mario Gonzalez-Carracedo, Mariano Hernandez, Hortense Slevogt, Thomas Grünewald

Supplementary Table S1. Description of *Clostridiodes difficile* clinical isolates analyzed in this study (ordered by frequent sequence types and isolation dates).

		Resistance phenotype[c]				Toxin genes[d]		Antibiotic resistance (AR) genes[e]									
Isolate ID	Sequence type (ST)[a]	Isolation date	MXF	RIF	DOX	Enterotoxin Cytotoxin (*tcdA*/*tcd*B)	Binary toxin (*cdt*A/*cdt*B)	*23S rRNA (C656T)*	*CDD-1, CDD-2*	*qacG*	*vanG cluster genes*	*ErmB; Erm(52)*	*gyrA (T82I)*	*rpoB (S485P, H502Y/N, R505K, I548M)*	*tet(M); tet(W); tet(40); tet(44)*	*APH(2'')-AAC(6'); aad(6); APH(3')-IIIa*	*cdeA*
Cd09	1	2010	R	R	S	+/+	+/+	+	+	+	+	+	+	+	-	-	-
Cd10	1	2011	R	R	S	+/+	+/+	+	+	+	+	+	+	+	-	-	-
Cd12	1	2011	R	R	S	+/+	+/+	+	+	+	+	+	+	+	-	-	-
Cd49	1	2012	R	R	S	+/+	+/+	+	+	+	+	+	-	+	-	-	-
Cd50	1	2012	R	R	S	+/+	+/+	+	+	+	+	+	+	+	-	-	-
Cd51	1	2012	R	R	S	+/+	+/+	+	+	+	+	+	+	+	-	-	-
Cd53	1	2012	R	R	S	+/+	+/+	+	+	+	+	+	+	+	-	-	-
Cd54	1	2012	R	R	S	+/+	+/+	+	+	+	+	+	+	+	-	-	-
Cd55	1	2012	R	R	S	+/+	+/+	+	+	+	+	+	+	+	-	-	-
Cd56	1	2012	R	R	S	+/+	+/+	+	+	+	+	+	+	+	-	-	-
Cd62	1	2014	R	R	S	+/+	+/+	+	+	+	+	+	+	+	-	-	-
Cd63	1	2015	R	R	S	+/+	+/+	+	+	+	+	+	+	+	-	-	-
Cd64	1	2015	R	R	S	+/+	+/+	+	+	+	+	+	+	+	-	-	-
Cd65	1	2015	R	R	S	+/+	+/+	+	+	+	+	+	+	+	-	-	-
Cd15	3	2008	R	S	S	+/+	+/+	+	+	+	+	+	+	-	-	-	-
Cd36	3	2008	R	S	S	+/+	+/+	+	+	+	+	+	+	-	-	-	-
Cd38	3	2009	R	S	S	+/+	-/-	+	+	+	+	+	+	-	-	-	-
Cd46	3	2011	R	R	S	+/+	+/+	+	+	+	+	+	+	-	-	-	-
Cd17	3	2012	R	S	S	+/+	+/+	+	+	+	+	+	+	-	-	-	-
Cd52	3	2012	R	S	S	+/+	+/+	+	+	+	+	+	+	-	-	-	-
Cd58	3	2014	R	S	S	+/+	+/+	+	+	+	+	+	+	-	-	-	-
Cd59	3	2014	R	R	S	+/+	+/+	+	+	+	+	+	+	-	-	-	-
Cd60	3	2014	R	S	S	+/+	+/+	+	+	+	+	+	+	-	-	-	-
Cd70	3	2015	R	S	S	+/+	+/+	+	+	+	+	+	+	-	+	-	-
Cd26	11	1997	S	S	R	+/+	+/+	+	+	+	-	-	-	-	+	-	-
Cd29	11	1997	R	R	R	+/+	+/+	+	+	+	+	+	+	+	+	+	+
Cd22	11	1998	R	R	R	+/-	+/-	+	+	+	-	-	-	-	-	-	-
Cd13	11	1998	S	S	S	+/+	+/+	+	+	+	-	+	-	-	+	+	-
Cd31	11	1998	R	R	R	+/+	+/+	+	+	+	+	+	+	+	+	+	+

Cd43	37	2009	R	R	R	+/+	-/-	+	+	+	-	+	+	+	+	+	-
Cd45	37	2010	R	R	R	+/+	-/-	+	+	+	-	+	+	+	+	+	-
Cd33	54	1997	R	S	R	+/+	+/+	+	+	+	+	+	+	-	+	-	+
Cd30	54	1997	R	R	R	+/+	+/+	+	+	+	+	+	-	+	+	-	+
Cd32	54	1998	R	R	R	+/+	+/+	+	+	+	+	+	+	+	+	+	+
Cd20	54	1998	S	S	R	+/+	+/+	+	+	+	+	+	+	-	+	-	+
Cd35	54	2008	R	S	R	+/+	+/+	+	+	+	+	+	+	-	+	-	+
Cd11	54	2008	R	R	R	+/+	+/+	+	+	+	+	+	+	+	+	+	+
Cd34	54	2008	R	R	R	+/+	+/+	+	+	+	+	+	+	+	+	+	+
Cd40	54	2009	R	S	R	+/+	+/+	+	+	+	+	+	+	-	+	-	+
Cd41	54	2009	R	S	R	+/+	-/-	+	+	+	+	+	+	-	+	-	+
Cd44	54	2009	R	R	R	+/+	+/+	+	+	+	+	+	+	+	+	+	+
Cd18	2	2012	S	S	S	+/+	+/+	+	+	+	+	-	-	-	-	-	-
Cd57	8	2014	S	S	S	+/+	+/+	+	+	+	+	-	-	-	-	-	-
Cd39	35	2009	R	R	R	+/+	-/-	+	+	+	+	+	+	+	+	-	-
Cd19	80	2012	S	S	S	+/+	+/+	+	+	+	+	-	-	-	-	-	-
Cd66	500	2015	R	R	S	+/+	+/+	+	+	+	+	+	+	+	+	+	-

[a]Multilocus sequence typing was performed under the *cdifficile* scheme (*adk, atpA, dxr, glyA, recA, sodA,*and *tpi*), available at PubMLST (https://pubmlst.org).

[c]Antibiotic resistance was tested for 6 antibiotics: moxifloxacin (MXF), rifampicin (RIF), doxycycline (DOX), daptomycin (DAP), metronidazole (MEZ), and vancomycin (VAN). All *C. difficile* isolates were classified as susceptible to DAP, MEZ and VAN. Legend: R, resistant; S, susceptible.

[d]Toxin genes were annotated by IonGAP platform (Baez-Ortega et al., 2015). Presence or absence of a specific gene is indicated by '+' or '-' symbol, respectively.

[e]AR genes were annotated by the CARD website (Alcock et al., 2023). Presence or absence of a specific gene (or gene variant) is indicated by '+' or '-' symbol, respectively. Other AR genes were also detected in a reduced number of isolates: *catP* (Cd11, Cd31, Cd32, Cd34, Cd35, Cd39, Cd44), *SAT-4* (Cd11, Cd29, Cd31, Cd32, Cd34, Cd44, Cd66), *clcD* (Cd39, Cd40, Cd43, Cd45), *mefH* (Cd39), and *nimJ* (Cd40).

Supplementary Table S2. Summary of the antibiotic resistance genes detected from WGS data.

Drug class (CARD)[a]	Genes detected[b]	No. clinical isolates	Total (%)
Macrolide, lincosamide	*23S rRNA (C656T)*	46	46 (100)
Carbapenem	*CDD-1*<br>*CDD-2*<br>*CDD-1 + CDD-2*	19<br>30<br>3	46 (100)
Disinfecting agents and antiseptics	*qacG*	46	46 (100)
Macrolide, Lincosamide, Streptogramin	*ErmB*<br>*Erm(52)*<br>*ErmB + Erm(52)*	39<br>9<br>7	41 (89,1)
Glycopeptide	*vanG cluster genes*	41	41 (89,1)
Fluoroquinolone	*gyrA(T82I)*<br>*cdeA*<br>*gyrA(T82I) + cdeA*	38<br>12<br>11	39 (84,8)
Rifamycin	*rpoB(S485P; H502Y)*<br>*rpoB(H502N; R505K)*<br>*rpoB(R505K; I548M)*	1<br>8<br>16	25 (54,3)
Tetracycline	*tet(M)*<br>*tet(M) + tet(W)*<br>*tet(M) + tet(40)*<br>*tet(M) + tet(W) + tet(44)*	15<br>2<br>1<br>1	19 (41,3)
Aminoglycoside	*AAC(6')-Ie-APH(2")-Ia*<br>*aad(6)*<br>*APH(3')-IIIa*<br>*aad(6) + APH(3')-IIIa*<br>*All genes*	2<br>1<br>1<br>5<br>1	10 (21,7)
Phenicol	*catP*	7	7 (15,2)
Nucleoside	*SAT-4*	7	7 (15,2)
Lincosamide, Streptogramin, Oxazolidinone, Phenicol; Pleuromutilin	*clcD*	4	4 (8,7)
Nitroimidazole	*nimJ*	1	1 (2,2)
Macrolide	*mefH*	1	1 (2,2)

[a]Annotated by the CARD website (Alcock et al., 2023).

[b]Antibiotic resistance genes:

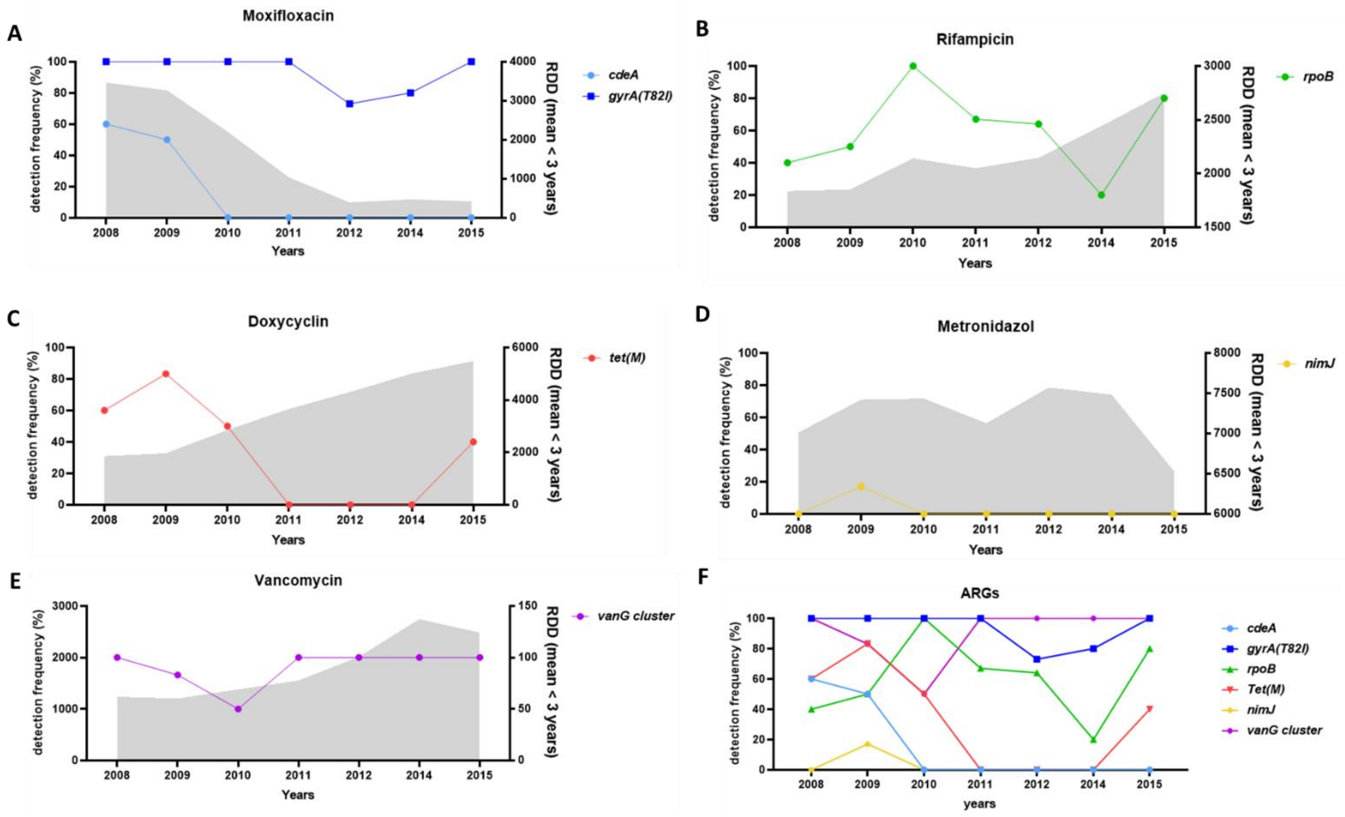

Supplementary Figure S1. Concordance between antibiotic recommended daily dosages (RDD) and resistance genes (ARG) frequencies.

A

B

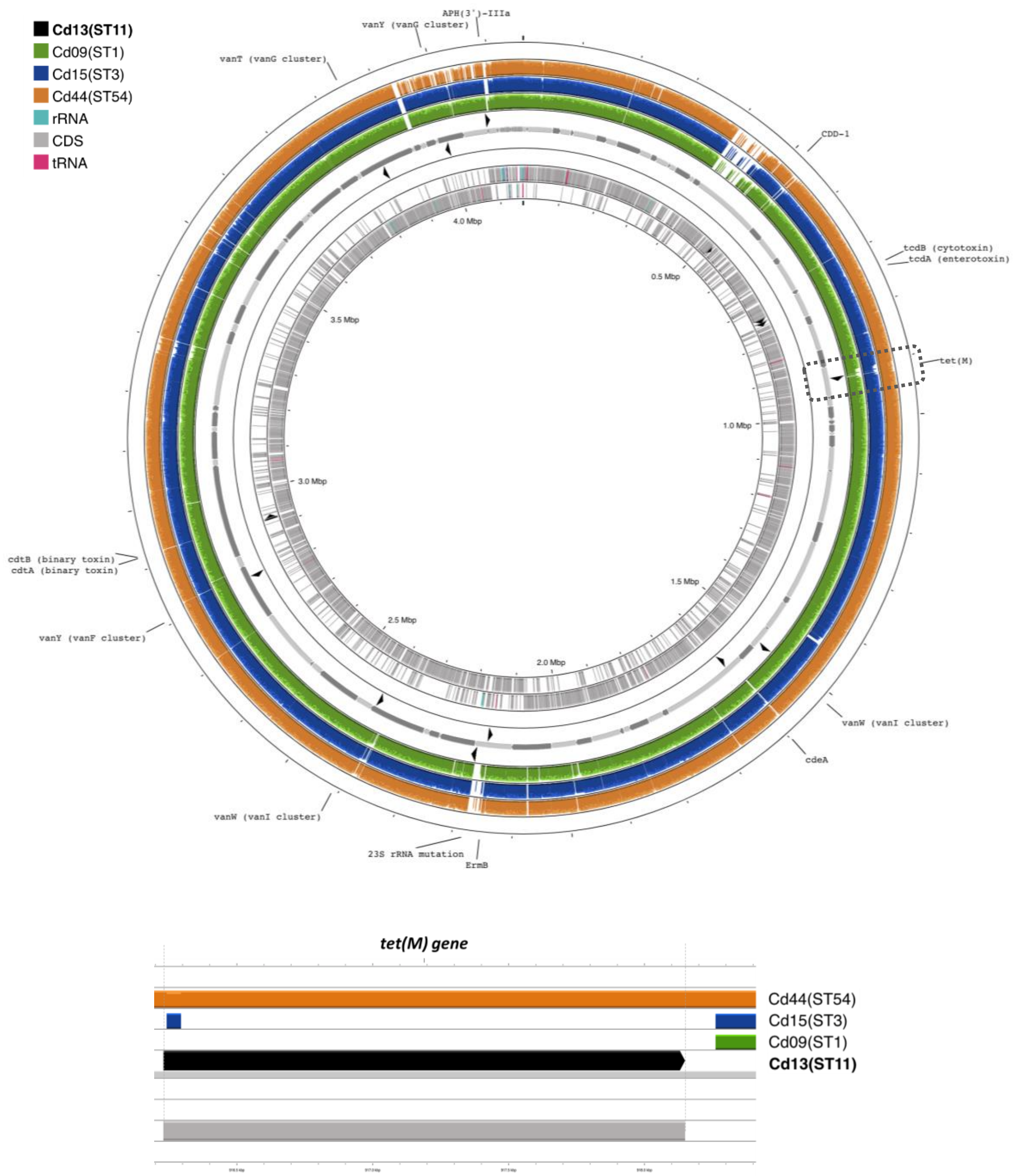

Supplementary Figure S2. Genome map of *C. difficile* strains. A) Circos plot of a ST11 representative strain (assembled contigs of Cd13 isolate), indicating annotated genes in both positive and negative DNA strands (inner rings). Outer coloured rings show BLAST alignments with ST1 (green), ST3 (blue) and ST54 (orange) representative strains. Antibiotic resistance and toxin genes are indicated (external layer). B) Physical location of the *tet*(M) gene in selected ST11 (black) and ST54 (orange) clinical isolates. This gene is absent in all ST1 (green) or ST3 (blue) analysed isolates.
